# Editorial: *Malassezia*: A Skin Commensal Yeast Impacting Both Health and Disease

**DOI:** 10.3389/fcimb.2021.659219

**Published:** 2021-02-19

**Authors:** Salomé LeibundGut-Landmann, Thomas L. Dawson

**Affiliations:** ^1^ Section of Immunology, Vetsuisse Faculty, University of Zürich, Zürich, Switzerland; ^2^ Institute of Experimental Immunology, University of Zürich, Zürich, Switzerland; ^3^ Skin Research Institute of Singapore, Agency for Science, Technology and Research, Singapore, Singapore; ^4^ Department of Drug Discovery, School of Pharmacy, Medical University of South Carolina, Charleston, SC, United States

**Keywords:** *Malassezia*, commensalism and symbiosis, pathogenicity determinants, skin pathologies, new tools

Over the last 30 years the term “microbiome” has grown exponentially in scientific discourse. Although the human-associated microbial community consists of bacteria, fungi, viruses, and archaea, the focus has been mainly on bacteria. Interest in the fungal communities, the mycobiome, has risen only more recently. In the skin, fungi represent 5%–10% of metagenomic sequences, versus less than 1% in mucosal epithelia (gut), and is dominated by a single fungal genus, *Malassezia*. Originally thought a single species, *Malassezia* is now known to span an entire clade comprising 18 diverse species and numerous functionally distinct strains. The lipid-dependent basidiomycetous yeast is found on all humans and warm-blooded vertebrates, and in ecosystems as diverse as deep marine environments. The high proportion of *Malassezia* in the skin microbiome makes understanding this fungus’ role at the intersection with the host, the environment, and the microbiota crucial. Importantly, beyond the commensal lifestyle on host skin, *Malassezia* is also associated with various pathological conditions, including dandruff, pityriasis versicolor, and common inflammatory skin disorders such as seborrheic dermatitis and atopic dermatitis. While in some cases disease is linked to the appearance of *Malassezia* hyphae and fungal overgrowth, the causal relationship between *Malassezia* and disease often remains unclear. Fungal pathogenicity is defined as a relationship where the fungus may directly harm the host by specific virulence factors, or indirectly *via* induction of a harmful host response. New pathogenic roles of *Malassezia* beyond the skin have been revealed as *Malassezia* is associated with Crohn’s disease and pancreatic ductal carcinoma. The present Special Topic of Frontiers in Fungal Pathogenesis makes a significant contribution to understanding more of the above-mentioned topics. The issue contains 13 diverse scientific contributions including 7 review and 6 primary research articles.

Vijaya Chandra et al. provide an up to date, comprehensive overview of *Malassezia* in human skin from early life to adulthood, including the importance of intact epithelial barriers and local immune system activity for stable colonization. However, how the *Malassezia*-host interaction transitions from a commensal to a pathogenic relationship remains a conundrum. The authors elaborate on whether *Malassezia* is cause or consequence in the multifaceted interaction with skin in health and under various dermatological conditions. They also discuss evidence for a protective, mutualistic, role of *Malassezia* in healthy skin through interaction with skin bacteria, especially atopic dermatitis-associated *S. aureus*, by restricting the bacteria’s pathogenicity. In addition to skin, *Malassezia* is found in other unrelated niches. The detection in healthy faecal samples by sequencing and culture indicates that viable *Malassezia* occupies the gastrointestinal niche. Spatz and Richard expand on the adaptive flexibility of *Malassezia* to grow under conditions that differ from skin with respect to lipid content, temperature, and oxygen. Fungal dysbiosis is a hallmark of intestinal inflammation and as such an increase in the abundance of *Malassezia* in the gut mycobiome is associated with IBD. Spatz and Richard also speculate on mechanisms for how *Malassezia* is involved in gut pathologies. Beyond the involvement in skin disease and to a lesser degree other barrier tissue, *Malassezia* causes severe systemic infections, especially among premature neonates and immunocompromised patients receiving parenteral nutrition. Although rare, their occurrence is underestimated and their outcome often fatal. Rhimi et al. review the latest about the epidemiology and pathogenesis of *Malassezia-*related fungemia.

*Malassezia* is not restricted to humans, but also found in diverse vertebrate species. Guillot and Bond discuss the relevance of *Malassezia* in veterinary dermatology, from original discovery of *M. pachydermatis* being an important canine otic pathogen to the recent discovery of *M. vespertilionis* in bats. They highlight how management of *Malassezia* otitis externa and *Malassezia* dermatitis, commonly presented to veterinarians in small animal practice, relies on both treating fungal overgrowth and restoring host immunity, as the latter is often a predisposing factor for fungal overgrowth. Successful management of *Malassezia*-related disease in humans and companion animals is hampered by poor diagnostics. Culture based isolation and enumeration of *Malassezia* remains challenging due to the lipid dependency. Saunte et al. review currently available diagnostic tools, including newer molecular methods, and treatment options for various skin pathologies. Identification of the involved *Malassezia* species is relevant for choosing the most effective antifungal drug, as different *Malassezia* spp. have varied antifungal susceptibility. The authors emphasize the need for standardization of species diagnostics and susceptibility testing. The challenges regarding robust and reproducible detection methods, treatment options and emerging resistance is also discussed by Rhimi et al. in the context of *Malassezia* fungemia and by Guillot and Bond in the context of *Malassezia*-mediated veterinary dermatitis and otitis.

Advances in *Malassezia* research are dependent on development of new tools for culture, detection, and genetic manipulation of the yeast, as well as for studying its pathogenicity and interaction with the mammalian host. Genome information is becoming available for an increasing number of species and strains, as summarized by Ianiri and Heitman and by Vijaya Chandra et al. Analysis of the available genomes contributed to resolving *Malassezia* taxonomy, shedding light on the evolutionary trajectory of *Malassezia* pathogenesis and niche adaptation, and enabling the identification of unknown genes and pathways linked to commensalism and/or pathogenicity. The availability of *Malassezia* genomes led to investigations considering emergence of *Malassezia* drug resistance. Park et al. assessed the molecular mechanisms that confer ketoconazole resistance, normally a highly effective fungistatic drug, in *M. restricta*. Comparative genome analysis of resistant strains isolated from dandruff patients suggests that multiplication of the genomic loci encoding genes involved in ergosterol synthesis and overexpression of drug efflux pumps are one mechanism underlying ketoconazole resistance in *M. restricta*. Genetic engineering of *Malassezia* was accomplished recently *via* agrobacterium-mediated transformation, as reviewed by Ianiri and Heitman. This powerful approach opens the door for both random insertional and targeted mutagenesis. The authors summarize the so far achieved mutants by either of the two approaches and discuss technical challenges and solutions. In addition, Goh et al. report generation of mCherry-expressing *M. furfur via* random insertional mutagenesis. Beyond fluorescently labelling yeast cells, this approach promises to be useful for overexpression of genes and the generation of insertional mutant libraries, which will help to deepen our understanding of *Malassezia* gene functions and host-microbes interactions.

Complementary to the new genetic tools, proteomic, metabolomic, and lipidomic approaches are essential for characterization of *Malassezia* species and strains in different environments. In this regard, Celis Ramírez et al. performed a lipidomic analysis and identified 18 lipid classes and 428 lipidic compounds, some of which segregate between different *Malassezia* species and may thus guide species discrimination and improve understanding of *Malassezia* lipid metabolism.

To study host-fungal interactions and assess antifungal immune responses, various models have become available. Studies with isolated cells in culture, including transformed keratinocyte and macrophage cell lines or primary cells isolated *ex vivo*, were recently complemented with approaches employing model hosts such as *Galleria mellonella* or experimental mice to explore the fungus-host interactions *in vivo*. In this Topic, Sparber et al. discuss the relevance of specific immune pathways for fungal control in barrier tissues. Type 17 immunity plays a central role in maintaining fungal homeostasis in healthy skin. However, IL-17 can exert pathological effects and exacerbate skin inflammation in barrier-disrupted skin. What determines the decision between protective and disease-promoting host response remains elusive. Also in this Topic, Corzo-León et al. describe an *ex vivo* human skin model, which provides a new and powerful approach to assess the host response to *Malassezia* of intact and fully differentiated human skin. The availability of diverse models opens the door for studying fungal pathogenesis and examining the functional role of putative virulence factors. As such, Vallhov et al. explored the host response to extracellular vesicles released from *M. sympodialis* and report activation of primary human keratinocytes, which strengthens the idea that extracellular vesicles play an important role in inter-organismal communication and initiation of the host response. The relevance of *Malassezia*-released compounds in the fungus-host interaction is further highlighted by Poh et al., which on the basis of genomic information explored secreted proteases in *M. furfur* and identified secreted aspartyl protease 1 (MfSAP1) as the dominant member of a family of 14 secreted proteases. They show that MfSAP1 is capable of degrading a wide range of human skin associated extracellular matrix proteins with important implication for cutaneous wound healing.

The diversity of contributions in this Special Topic shows that we are only scratching the surface to understanding *Malassezia* biology and the complexities of *Malassezia*-host interactions. A causative relationship between the fungus and various pathologies remains a matter of debate and the mechanism of pathogenesis unclear. Increased understanding of host- and microbe-specific interactions should lead to identification of key factors that maintain healthy skin homeostasis or, in turn, initiate pathogenic changes. Recent developments have expanded our understanding of *Malassezia*’s role in the skin microbiome, with a focus on its multiple roles as commensal, pathogen, and protector through interactions with the host and inter-kingdom interactions. These approaches are leading towards development of new therapeutic targets and treatment options.

The guest editors would like to thank all the authors who submitted their work to the Special Topic, and all the reviewers for their hard work in completing timely and constructive reviews. Special thanks also go to the Editor-in-Chiefs and members of the editorial team for their support during the editing process.

## Author Contributions

SL-L drafted the manuscript. All authors contributed to the article and approved the submitted version.

## Funding

The authors would like to acknowledge funding from the A*STAR Industry alignment fund (H18/01/a0/016, to TLD) and the Swiss National Science Foundation (310030_189255, to SL-L).

## Conflict of Interest

The authors declare that the research was conducted in the absence of any commercial or financial relationships that could be construed as a potential conflict of interest.

